# Correlation Between Schizophrenia and Coronavirus Disease in North Sumatera, Indonesia: A Correlative Analytical Study

**DOI:** 10.3389/fpsyt.2022.896623

**Published:** 2022-05-30

**Authors:** Mustafa M. Amin, Richie Futrawan, Muhammad Surya Husada

**Affiliations:** Department of Psychiatry, Faculty of Medicine, Universitas Sumatera Utara, Medan, Indonesia

**Keywords:** schizophrenia, age, coronavirus disease, COVID-19, Indonesia

## Abstract

**Background:**

In the first quarter of 2020, two cases of coronavirus disease (COVID-19) were reported in Indonesia, approximately 4 months after the first case was reported in China. The numbers continued to increase following the introduction of many variants of the virus. The pandemic may have an impact on the community's mental health, particularly on those with mental illnesses. Therefore, this study aimed to determine the correlation between schizophrenia and COVID-19 based on demographic characteristics.

**Methods:**

This nominal-nominal and numerical-nominal correlative analytical study used a cross-sectional approach and was conducted at a psychiatric hospital in North Sumatra. The sample population consisted of 48 patients and 48 healthy controls, who were selected using a non-probability consecutive sampling method.

**Results:**

The analysis showed that there were correlations between schizophrenia and COVID-19 (*r* = 0.417, *p* < 0.001) and between the age of patients with schizophrenia and COVID-19 with (*r* = 0.544).

**Conclusions:**

COVID-19 is correlated with schizophrenia and the age of patients with schizophrenia. We recommend that patients with schizophrenia follow the same health guidelines as the clinical high-risk group for COVID-19 and receive the same treatment. Physicians that treat patients with COVID-19 should pay close attention to those with schizophrenia because they may underestimate their condition.

## Introduction

In Indonesia, two cases of coronavirus disease (COVID-19) were announced on March 2, 2020, approximately 4 months after it was first identified in China. Subsequently, two other confirmed patients were discovered on March 6, 2020, and the numbers have continued to increase (1). At the end of March 2022, there were approximately 6 million confirmed cases of COVID-19 in Indonesia (2).

This situation has led to the development of several studies on the effects of the pandemic on the mental health of the general community, especially in patients with mental disorders (3). During the pandemic in Indonesia, two studies were conducted on mental health. The first study by Kaligis et al. (4) concluded that anxiety symptoms were the most reported symptoms of the participants. A similar result was reported by Izzatika et al. (5), who found that 34.6% of the study population experienced anxiety. Previous studies have shown that the stress associated with the virus and its preventive measures have a negative effect on mental health, particularly among people with schizophrenia. Furthermore, viral infection can worsen the symptoms experienced in schizophrenia because it is associated with psychotic symptoms through immune mechanisms (6), particularly cytokines. Schizophrenia is linked to disruption of the cytokine milieu and a tendency for the development of pro-inflammatory cytokines (7). The pandemic has had an unprecedented impact on several countries worldwide, and preventive efforts have been disproportionately burdened by the presence of schizophrenia and other disorders (8).

Mechanisms related to the association between coronavirus infection and mental disorders have revealed the involvement of neuroimmune networks. Furthermore, elevated cytokine levels have been observed in some psychiatric disorders as a sign of immunity, which is also common in patients with COVID-19. Dissolved cytokines in the brain or their corresponding local alteration levels can affect the synthesis, release, and reuptake of several neurotransmitters, including monoamines, such as dopamine, norepinephrine, and serotonin. Alterations in metabolism are also involved in the pathophysiology of various psychiatric disorders. Changes in cytokine levels cause disturbances in metabolism, which trigger behavioral deficits. Therefore, the immune system can be assumed to be the link between COVID-19 infection and mental health disorders (9). There are limited studies on the correlation between schizophrenia and the coronavirus pandemic; hence, this study is expected to provide information for clinicians, patients, families, and the community. This study aimed to investigate the implications of the global pandemic related to the increased risk of infection and poor outcomes among patients with schizophrenia, as well as the anticipated adverse mental health consequences of the disorder.

## Methods

### Ethics Statements

All participants provided written informed consent after they were given a detailed and clear explanation of the study process. This study was approved by the Research Ethics Committee of the Universitas Sumatera Utara (Reference Number: 838/KEP/USU/2021).

### Study Design and Population

This was a correlative analytical study with a cross-sectional approach that assessed the correlation between people with schizophrenia and COVID-19. The independent variables were age, sex, and schizophrenia, whereas the dependent variable was COVID-19. Furthermore, the study was conducted at the Psychiatric Hospital of North Sumatera Province, Medan for 5 months, i.e., between September 2021 and January 2022.

The number of confirmed cases was the lowest according to the COVID-19 National Taskforce during these months. The study was conducted during a pandemic; however, the government never implemented a lockdown policy. They only had a regulation to restrict activity for the community.

The sample population consisted of patients with schizophrenia and healthy controls living around the research location, who were selected using a consecutive sampling method. The inclusion criteria for the schizophrenia group were people with schizophrenia regardless of the duration of their illness based on the International Classification of Disease and Related Health Problems Tenth edition; those with a Positive and Negative Syndrome Scale score of 80120 that was measured when they first came to the hospital; those aged 18–45 years; and those who were cooperative and willing to be interviewed. The exclusion criteria were a history of other psychiatric disorders, neurological diseases, endocrine disorders, autoimmune diseases, alcohol use, and other addictive substance use (except nicotine and caffeine). The healthy control group comprised people aged 18–45 years with no psychiatric disorders after screening based on the Mini-International Neuropsychiatric Interview and those who were cooperative and willing to be interviewed. A history of family psychiatric disorders, neurological diseases, autoimmune diseases, endocrine disorders, and alcohol and other addictive substance use were also exclusion criteria for this group.

### Data Collection

A nasal swab specimen was collected from each sample, after which laboratory tests were performed. The antigen rapid diagnostic test (Ag-RDT) examination technique, which involves specimen collection and examination, was performed by trained health personnel as well as a laboratory analyst at the North Sumatera Psychiatric Hospital. They strictly adhered to standard procedures based on the type of Ag-RDT used, which can be in the form of a nasal swab. Universal precautions to prevent disease transmission were followed before the specimens were collected.

### Sample Size Calculation

The population size required was calculated using the equation below (10):


$$n={\left\{\frac{Z\text{α}+Z\text{β}}{0.5\ln\left[\frac{\left(1+r\right)}{\left(1-r\right)}\right]}\right\}}^{2}+3\;$$

*n* = minimum sample size

α = type I error, set at 5%

Zα = alpha standard value (1.96), two-way hypothesis

β = type II error, set at 20%

Zβ = beta standard value (0.84)

*r* = the minimum correlation that is considered significant was determined (0.4)

Subsequently, a value of 47.49 was obtained, which was rounded to 48; hence, the sample population consisted of 48 patients with schizophrenia and 48 healthy controls.

### Data and Statistical Analyses

After collecting all data from patients with schizophrenia and the controls, i.e., age, sex, and COVID-19 status, data processing was performed in several stages: (1) editing, a step to examine the completeness of the data obtained through interviews; (2) coding, classifying the answers based on their type; (3) tabulation, entering the data into a table based on the variables studied; and 4) data analysis. Categorical data are presented as number (*n*) and percentage (%), while numerical variables are presented as mean and standard deviation. No normality test was conducted before the data were analyzed using correlation tests. The nominal-nominal correlation test is the contingency coefficient test, whereas the numerical-nominal correlation test is the eta test. When data were normally distributed, the Shapiro–Wilk test was used to determine the median because the number of samples was <50 (*p* < 0.05). Data processing and analysis were performed using the Statistical Package for Social Sciences (SPSS) software, version 24 (IBM Corp.).

## Results

The age, sex, and COVID-19 status of participants in the schizophrenia and control groups are shown in Table 1. Each group had 30 men, accounting for 62.5% of the total population. Thirty-four patients in the schizophrenia group (70.8%) were positive for COVID-19, whereas 36 of participants in the control group did not have COVID-19. The schizophrenia group had a median age of 25 (range, 18–41) years, while the control group had a median age of 28.50 (range, 25–38) years. The result of the eta test revealed an *r*-value of 0.544.

**Table 1:** Demographic characteristics of patients with schizophrenia and the controls.

**Variable (n1 + n2 = 48)**	**Median (min–max) *n* (%)**
Patients with schizophrenia, age	25 (18–41)
Controls, age	28.50 (25–38)
**Patients with schizophrenia, sex**	
-Male	30 (62.5)
-Female	18 (37.5)
**Controls, sex**	
-Male	30 (62.5)
-Female	18 (37.5)
**Patients with schizophrenia**	
-Positive for COVID-19	34 (70.8)
-Negative for COVID-19	14 (29.2)
**Controls**	
-Positive for COVID-19	12 (25)
-Negative for COVID-19	36 (75)

*COVID-19, coronavirus disease; min, minimum; max, maximum*.

Table 2 shows that there was a correlation between the age of patients with schizophrenia and COVID-19 (*r* = 0.544). Consequently, another eta correlation test was performed to determine the relationship between the age of the control group and COVID-19, an *r*-value of 0.243 was recorded along with a type 1 error or an error value of 0.05.

**Table 2:** Correlation of the age of the patients with schizophrenia and the controls with COVID-19.

**Variable**	**Median (min–max)**	***n***	***r*-value**
**Patients with schizophrenia**			
Age	25 (18-41)		0.544*
**COVID-19**			
Positive		34	
Negative		14	
**Controls**			
Age	28.50 (25–38)		0.243*
**COVID-19**			
Positive		12	
Negative		36	

*Eta correlation test.

*COVID-19, coronavirus disease; min, minimum; max, maximum*.

Table 3 shows the relationship between the sex of patients with schizophrenia and COVID-19; 22 men and 12 women tested positive for the virus. This indicated that there was no correlation between the two variables (*r* = 0.071, *p* = 0.623). A similar result was found in the control group, where 20 men and 16 women tested negative for the virus. This indicated that there was no correlation between the two variables (*r* = 0.241, *p* = 0.085).

**Table 3:** Correlation of the sex of the patients with schizophrenia and the controls with COVID-19.

**Variable**	**Positive for COVID-19 (*n*)**	**Negative for COVID-19 (*n*)**	***r*-value**	***p*-value**
**Patients with schizophrenia**				
**Sex**				
Male	22	8	0.071	0.623*
Female	12	6		
**Controls**				
**Sex**				
Male	10	20	0.241	0.085
Female	2	16		

*Contingency coefficient test.

*COVID-19, coronavirus disease*.

Table 4 shows the relationship between schizophrenia and COVID-19; 34 patients with schizophrenia tested positive for the virus, whereas 36 control samples were negative. Furthermore, the statistical test revealed a correlation between schizophrenia and COVID-19 (*r* = 0.417, *p* < 0.001).

**Table 4:** Correlation of schizophrenia and non-schizophrenia with COVID-19.

**Variable**	**Positive for COVID-19**	**Negative for COVID-19**	***r*-value**	***p*-value**
Schizophrenia	34	14	0.417	<0.001*
Controls	12	36		

*Contingency coefficient test.

*COVID-19, coronavirus disease*.

## Discussion

We discovered a correlation between the age of patients with schizophrenia and COVID-19. This finding is in line with the result of a study from 2020 performed by Hu et al. in China that compared 13,783 data points recorded in January 2020. Their study revealed that COVID-19 increased the risk of schizophrenia among people within the age range of 29 to 50 years at the first onset of psychosis during the pandemic. They thought it was due to older people being more prone to COVID-19, which could increase the risk of schizophrenia. The emergence of COVID-19 could make older adults emotionally vulnerable, and too much information could put strain on each person's nerves. It may also increase the risk of severe mental illnesses, such as schizophrenia (11). Age is related to mental health status, including schizophrenia, and old age affects the incidence of and morbidity and mortality due to COVID-19. Previous studies have shown that patients with COVID-10 had a more robust host innate response to viral infection than others without COVID-19 of the same age. They also showed increased differential expression of genes associated with inflammation, while that of beta-interferon I was reduced. Furthermore, age-dependent defects in T- and B-cell function, as well as overproduction of type 2 cytokines, could lead to a deficiency in controlling viral replication. These conditions can also cause a prolonged pro-inflammatory response, leading to poor outcomes (12).

We found that COVID-19 was not correlated with the sex of the patients with schizophrenia and the controls. This finding is contradicted by Wang et al. (13), who analyzed secondary data from 360 hospitals and 317,000 providers in the United States. Their study revealed that compared to their male counterparts, female patients with schizophrenia are at a higher risk of experiencing COVID-19, after their age, ethnicity, and medical comorbidities were analyzed (13). Physical distancing can worsen female patients' symptoms, and they felt that their lives were more stressful than male patients' lives during the pandemic (14); the latter could induce symptoms of schizophrenia. Moyser postulated that women might be doing unpaid family work that their households would have previously outsourced to the paid economy or with which their households would have previously received help from extended family or friends due to the closure of daycare, schools, and businesses, such as restaurants and dry cleaners (14). These situations may lead to stressful events that can precipitate mental illness.

Furthermore, there was a correlation between schizophrenia and COVID-19. This result is in line with the findings of Wang et al. (13), who reported that patients with schizophrenia were more susceptible to viral infection. A possible mechanism for the correlation between COVID-19 infection and mental health outcomes is involvement of the neuroimmune network. This finding suggests that elevated levels of various cytokines can be observed in some psychiatric disorders, which also serve as immune markers for COVID-19. The presence of dissolved cytokines in the brain or their corresponding local alteration levels can affect the synthesis, release, and reuptake of several neurotransmitters, including monoamines, such as dopamine, norepinephrine, and serotonin (15). Changes in their levels can cause disturbances in metabolism, thereby triggering behavioral deficits. Therefore, the immune system can be used as a link between COVID-19 and mental health disorders. Several studies have shown that cytokines play essential roles in learning and memory. Moreover, under healthy conditions, increased expressions of interleukin (IL)-1β, IL-1, IL-6, and IL-18 receptor antagonists occur in the hippocampus during long-term potentiation (LTP), which is believed to underlie certain forms of brain function (16–18). It is thought that IL-1β is associated with LTP maintenance, learning acquisition, and memory consolidation, which indicates that IL-6 has the opposite effect. Peripheral and central diseases are characterized by elevated levels of cerebral IL-1β and IL-6 levels, which inhibit synaptic plasticity, learning, and memory (19). High levels of IL-6 are often found in the blood of patients infected with COVID-19. It is also present in the central nervous system of cytokeratin 18 promoter (K18-hACE2) transgenic mice infected with coronavirus (20, 21).

Signs of a peripheral inflammatory response in schizophrenia are indicated by elevated serum/plasma levels of certain pro-inflammatory factors, including prostaglandin E2 and C-reactive protein, as well as some pro-inflammatory cytokines, such as IL-1β, IL-6, IL-8, and tumor necrosis factor-α. Additionally, the peripheral inflammatory response in patients with schizophrenia involves aberrant monocytes, which are a primary source of these molecules. A previous study reported a significant increase in the absolute or relative numbers of monocytes and white blood cells in patients with this disorder (22). Subsequently, there is an imbalance between pro-inflammatory and anti-inflammatory cytokines associated with psychiatric disorders, such as schizophrenia. Data from a previous immunological study revealed elevated levels of peripheral inflammatory markers in patients with schizophrenia (23).

Political and health authorities must pay attention to the mental health of infected and uninfected individuals during a pandemic. In addition, several preventive and treatment strategies must be developed. Some strategies that were developed were as follows: (1) investing in media campaigns, i.e., federal and state leaders must invest in public health campaigns that normalize discomfort, destigmatize mental health issues, especially schizophrenia, promote self-care, convey effective preventative and treatment measures, and make mental health services more accessible (24). In Indonesia, mental health promotion is still not comprehensive; many promotions were done by the Indonesian Psychiatric Association or by the psychiatrists themselves. This issue needs to be taken seriously to reduce the stigma of mental illnesses, especially schizophrenia. However, the availability of psychotropic medication is limited in primary care services, so patients with schizophrenia still need to come to the hospital to receive treatment. (2) Increasing the number of people who are screened for mental illness and schizophrenia. Given the prevalence of psychological distress during the pandemic, widespread mental health screenings should be implemented. Vaccine administration provides an excellent universal context, and mental health screening should be initiated as part of return-to-work and return-to-school programs. Specific high-risk professions should also be screened, i.e., frontline workers, those with poor incomes, and those who are more socially isolated, who are disproportionately affected by the mental health implications of COVID-19 (24). Mental health screening in Indonesia, especially for detecting schizophrenia, is rare. This is due to the lack of physicians who have been trained to use a questionnaire for screening mental illnesses and the high number of patients visiting the primary care unit; as a result, physicians do not have time to perform screening. (3) Focus on the most critical interventions, i.e., establish population-level measures to minimize distress, promote resilience, and provide specialized services for people at highest risk of distress (24). Psychiatrist is not well distributed in Indonesia, and many of them stay in urban areas rather than rural areas. If the distribution problem can be solved, intervention for people with mental illnesses, especially schizophrenia, will be easier. (4) Expanding capacity, i.e., prior to the epidemic, the mental health treatment system was already struggling to satisfy the mental health requirements of many countries. Now is the time to invest in the workforce of social workers, psychiatric nurse practitioners, psychologists, master's-level therapists, psychiatrists, and peer counselors (24). As aforementioned, the number of professionals dealing with mental health issues in Indonesia is limited. Psychiatry is not a popular choice compared with internal medicine, surgery, child health, and obstetrics/gynecology. There are not many psychologists, psychiatric nurse practitioners, or peer counselors available, even in urban areas. In the future, the Indonesian government should pay more attention to providing scholarships or incentives to people who want to study in this field of service. (5) Make mental health surveillance and research a top priority; this pandemic is unlike any other, and it is unclear how the mental health consequences will play out over time. Real-time population mental health monitoring and the collection of high-quality longitudinal and representative data must be emphasized to identify risks and understand longer-term trajectories of distress and resilience. At the population and individual levels, ongoing research should examine communication initiatives, screening programs, systems of care workforce development, and new and existing interventions. These data, especially on the integration of mental and physical healthcare during and after the epidemic, must guide our responses to future crises (24). Psychiatric research is still not a top priority in Indonesia, and the top national research priority is still food and energy. Health research is still focusing on stunting and reducing the number of deaths of babies and mothers during delivery. Things to look forward to in the future include approaching the government to increase awareness of the importance of mental health and significantly increasing research funds for psychiatry, especially for schizophrenia.

Poor mental health is associated with a reduced life span and higher economic burden. In addition to the urgent and fundamental task of saving lives during the COVID-19 pandemic, psychiatric care needs to be provided on time. Several protocols must be implemented to minimize mental problems that occur during infection and after hospitalization. Moreover, a study evaluating the impact of isolation on mental health during the pandemic is essential because it can serve as a guide for the development of new strategies in other critical situations (25). The approach used for psychoneuroimmunology in COVID-19 needs to complement that of social science because it provides a better understanding of how to overcome the disease. Future studies should test the hypotheses outlined, as this is expected to help reduce the impact of COVID-19 on mental health. Some strategies can be implemented to prevent COVID-19 in the schizophrenia population; for example, schizophrenia patients should follow the same health guidelines as clinical high-risk groups for COVID-19 and receive the same treatment; patients with schizophrenia should receive additional attention from the general practitioners treating COVID-19 patients since they may underestimate or have trouble describing respiratory symptoms; and antipsychotic medication adherence should be promoted and monitored by professionals and families (26).

As far as we are concerned, the strength of this study is that there are no studies with similar methods and measuring tools that were conducted in Medan and Indonesia in general. This study is the first to explore the correlation between schizophrenia and COVID-19. However, it has several limitations, such as the small sample size owing to the level 3 and 4 regulations for the implementation of community activity restrictions by the government. This study was also performed at one health center because of limited human resources; hence, the results do not represent the national population. We recommend that (1) future research should repeat the study using a larger sample size, (2) samples should be collected from private hospitals too, and (3) data from other Asian nations should be combined.

## Conclusions

The COVID-19 pandemic, which has affected several countries, has revealed that people with schizophrenia are more susceptible to the virus. This result is in line with findings from several studies showing that patients with schizophrenia have lower immunity than healthy people. There is also the impression that such patients often receive less attention from the government regarding their physical health due to stigmatization. Therefore, special regulations must be implemented to ensure that people with schizophrenia receive vaccines first to increase their immunity.

## Data Availability Statement

The original contributions presented in the study are included in the article/supplementary material, further inquiries can be directed to the corresponding author/s.

## Ethics Statement

The studies involving human participants were reviewed and approved by Research Ethical Committee of Universitas Sumatera Utara. The patients/participants provided their written informed consent to participate in this study.

## Author Contributions

All authors listed have made a substantial, direct, and intellectual contribution to the work and approved it for publication.

## Conflict of Interest

The authors declare that the research was conducted in the absence of any commercial or financial relationships that could be construed as a potential conflict of interest.

## Publisher's Note

All claims expressed in this article are solely those of the authors and do not necessarily represent those of their affiliated organizations, or those of the publisher, the editors and the reviewers. Any product that may be evaluated in this article, or claim that may be made by its manufacturer, is not guaranteed or endorsed by the publisher.
